# Short-term fish oil supplementation applied in presymptomatic stage of Alzheimer's disease enhances microglial/macrophage barrier and prevents neuritic dystrophy in parietal cortex of 5xFAD mouse model

**DOI:** 10.1371/journal.pone.0216726

**Published:** 2019-05-16

**Authors:** Milena Jović, Nataša Lončarević-Vasiljković, Sanja Ivković, Jelena Dinić, Desanka Milanović, Berislav Zlokovic, Selma Kanazir

**Affiliations:** 1 Department of Neurobiology, Institute for Biological Research ‘Sinisa Stankovic’, University of Belgrade, Belgrade, Serbia; 2 Department of Physiology and Neuroscience, Zilkha Neurogenetic Institute, Keck School of Medicine, University of Southern California, Los Angeles, CA, United States of America; Torrey Pines Institute for Molecular Studies, UNITED STATES

## Abstract

Dystrophic neurites and activated microglia are one of the main neuropathological characteristics of Alzheimer's disease (AD). Although the use of supplements with omega-3 fatty acids has been associated with reduced risk and lessened AD pathology, it still remains elusive whether such a treatment could affect dystrophic neurites (DNs) formation and microglia/macrophage behavior in the early phase of disease. We analyzed the effects of short-term (3 weeks) fish oil supplementation on DNs formation, tau hyperphosphorylation, Amyloid-beta peptide 1–42 (Aβ42) levels and microglial/macrophage response to AD pathology in the parietal cortex of 4-month-old 5xFAD mice, a mouse model of AD. The present study shows for the first time that short-term FO supplementation applied in presymptomatic stage of AD, alters the behaviour of microglia/macrophages prompting them to establish a physical barrier around amyloid plaques. This barrier significantly suppresses DNs formation through the reduction of both Aβ content and tau hyperphosphorylation. Moreover, the short-term FO treatment neither suppresses inflammation nor enhances phagocytic properties of microglia/macrophages in the response to Aβ pathology, the effects most commonly attributed to the fish oil supplementation. Our findings suggest that fish oil consumption may play an important role in modulating microglial/macrophage response and ameliorating the AD pathology in presymptomatic stage of Alzheimer's disease.

## Introduction

Alzheimer's disease (AD) is the most prevalent neurodegenerative disease and the main form of progressive dementia in elderly [1]. It is characterized by cognitive deficits that initially affect learning and memory, leading to the overall cognitive decline as the disease progresses. Although the AD clinical symptoms such as memory loss and impaired cognition occur in the older age it is estimated that the pathological processes underlying AD begin to develop even a few decades earlier [2]. Therefore, developing treatments for the presymptomatic phase of the disease is gaining in importance.

Defining feature of AD pathology is the formation of amyloid plaques, structures composed of fibrillar amyloid-β organized in a β-sheet conformation. Amyloid-β protein (Aβ) is the pivotal mediator of neuronal cell loss in the AD brain [3–5] with Aβ42 found to be the most toxic form [6, 7]. Plaques are mostly encircled by a halo of diffuse Aβ42, surrounded by dystrophic neurites (DNs) and activated glia [5, 8]. The areas of diffuse Aβ42 were recently named “hot-spots” and they are considered as the zones of greater neuro-toxicity [9]. Dystrophic neurites surrounding the plaques represent the initial phase of neurodegeneration. These varicose and beaded neurites are characterized by extensive microtubule disruption, due to the abnormal hyperphosphorylation of tau protein, and inhibited anterograde and retrograde trafficking [10, 11].

Prolonged microglial activation has an important role in the pathogenesis of AD [12, 13]. The distinctive feature of microglial behavior in the AD brain is their noticeable clustering around fibrillar Aβ deposits, which are also in close proximity to dystrophic neurites [14, 15]. However, the exact role of microglia in AD progression is still controversial.

The post-mortem studies as well as functional imaging studies have revealed that the changes in the parietal lobe, besides those in the temporal and frontal cortex, contribute to the AD progression [16–18]. Furthermore, these changes are gaining focus as a potential marker for the early diagnosis of AD in accordance with the ‘last-developed–first-atrophied’ concept [19]. The higher cognitive association areas, which mature after the primary areas, show the first signs of functional decline and grey matter atrophy [19, 20]. The involvement of the parietal cortex during the early phase of AD is likely due to its strong connectivity with other brain areas, a role in memory retrieval [21] and the wide range of cognitive functions relying on its proper functioning [22].

It has been shown that specific components of our diet can affect the progression of AD [23, 24] and docosahexaenoic acid (DHA) is one of them. The levels of DHA are decreased in brains and serums of AD patients [25, 26], and ω-3 polyunsaturated fatty acids (ω-3 PUFA) supplementation had become of a major interest as potential adjunctive treatment for AD patients. In humans, positive effects of DHA or eicosapentaenoic acid (EPA) supplementation have been seen only in cognitively healthy participants and participants with mild cognitive impairment [27]. Numerous clinical trials on patients with established AD revealed that EPA and DHA supplementation did not slow the rate of cognitive decline compared to the placebo [28]. In mouse AD models oral intake of PUFAs and fish oil (FO), a rich source of essential PUFAs—DHA and EPA has been associated with the decreased amyloid deposition in the early stages of AD and a reduced risk of AD if applied before the onset of AD pathology [29–32]. However, the effect of FO supplementation on neuritic pathology in the early phase of the disease has not been addressed so far.

Considering that the appearance of the DNs represents an early event that precedes neuronal loss, we aimed to examine whether the modification in dietary consumption via the addition of FO, in the presymptomatic phase of AD pathology, could attenuate or even prevent the progression of the DNs formation. To this purpose we have chosen the 5xFAD mouse model [33], which displays a rapid deposition of Aβ and a massive loss of pyramidal neurons [34] with the initial structural insults of neuronal cells appearing between 4 to 6 months of age [35]. We analyzed the effects of the short-term (3 weeks) FO treatment on DNs formation, p-Tau (Ser416) and Aβ42 levels, and on the distribution, number and localization of microglia/macrophages in 4-month-old 5xFAD mice.

## Materials and methods

### Animals and procedures

#### Ethics statement

All animal procedures were in compliance with Directive (2010/63/EU) on the protection of animals used for experimental and other scientific purposes and were approved by the Ethical Committee for the Use of Laboratory Animals (resolution No 01-06/13) of the Institute for Biological Research “Sinisa Stankovic”, University of Belgrade. Minimal numbers of animals were used and all efforts were made to minimize animal suffering.

#### Experimental animals and fish oil treatment

Three months old 5xFAD mice (B6SJL-Tg (APPSwFlLon, PSEN1*M146L *L286V) 6799Vas) from The Jackson Laboratory, stock number: 34840-JAX/5XFAD [33] were used for all experiments. Mice were divided into two experimental groups: control group (marked as 5xFAD) and treated group (marked as 5xFAD FO). The fish oil used in current study was ‘Omega 3’, commercial fish oil for human consumption standardized to 12% DHA and 18% EPA (Dietpharm-Atlantic Group, Croatia). Treated animals received 100 μl of commercial fish oil per day by oral gavage as previously described [30]. The control group received the same volume of the water, also by oral gavage. The animals were housed under standard conditions (23 ± 2°C, 60–70% relative humidity, 12 h light/dark cycles, with the lights switched on at 07:00; free access to food and water; n = 3 per cage). The discomfort following oral gavage, capture, handling and restraint were minimized, due to the refinements in housing, husbandry and care and good practice for substance administration.

#### Sample preparation

Following 3 weeks of FO treatment, when reached age of 3 months and 3 weeks, animals were anesthetized with ketamine (Ketamidor, Richter pharm, Germany) in dosages of 250mg/kg. Mice were perfused with ice-cold phosphate-buffered saline (PBS), brains were harvested, and the cortex was extracted, quickly frozen, and stored at −80°C until use for RNA and protein analysis.

### RNA and protein analysis

#### Quantitative real-time PCR

Quantitative real-time PCR was performed using the ABI Prism 7700 Sequence Detection System (PE Applied Biosystems) to analyze the induction of inflammatory cytokine mRNAs, following the manufacturer’s instructions. Briefly, total RNA was extracted from the cortical tissue using TRIzol reagent. For cDNA synthesis, 1μg of total RNA was transcribed with TaqMan reverse transcription reagents using random hexamers. The cDNA was stored at −20°C until further use. Relative quantification of TNFα and IL-1β mRNAs was performed by real-time RT-PCR with the Applied Biosystems TaqManGene Expression Assays (TNFα - Cat No. 4331182, IL-1β –Cat.No. 4351372; Thermo Fisher Scientific). GAPDH was included as an endogenous control to correct for differences in interassay amplification efficiency (ID Rn99999916_s1; Applied Biosystems). Each sample was run in triplicate and the mean values of each Ct were used for further calculations. Quantification was performed by the 2 ^−ΔΔCt^ method. The results obtained by RT-PCR were analyzed in RQ Study add-on software for the 7700 v 1.1 SDS instrument, with a confidence level of 95% (P < 0.05).

#### Enzyme-linked immunosorbent assay

Enzyme-linked immunosorbent assay (ELISA) was used to quantify the cortical level of TNF-α. TNF-α ELISA kit (ab100747, Abcam) was used according to the manufacturer’s recommendations. Tissue was homogenized on ice in the extraction buffer recommended by the manufacturer (100 mM Tris, pH 7.4, 150 mM NaCl, 1 mM EGTA, 1 mM EDTA, 1% Triton X-100, 0.5% sodium deoxycholate) with 1 mg/mL of protease inhibitor cocktail (cOmplete, Sigma-Aldrich) and 0.01 mg/mL of phosphatase inhibitor cocktail (P5726, Sigma-Aldrich). The protein concentrations were determined using a Micro BCA protein assay kit (Pierce, Rockford, IL). The absorbance at 450 nm was measured with an iMark Microplate Absorbance Reader (Bio-Rad).

#### Western blot analysis

The extracted cortical tissue was homogenized in 10 vol (w/v) of RIPA buffer (50 mM Tris-Cl pH 7.5, 150 mM NaCl, 1% NP-40, 0.1% SDS, 10 mM EDTA pH 8.0, 10 mM EGTA pH 7.2, 0.5% Triton X-100), followed by centrifugation (21 000 rcf, 30 min, 4ºC); the supernatant was collected. Protein concentrations were determined using the Micro BCA Protein Assay Kit (Pierce Biotechnology). Equal amounts of proteins (30 μg per lane) were loaded and separated by 10% SDS acrylamide gel electrophoresis and blotted onto nitrocellulose membranes (Amersham Bioscience). The membranes were blocked at room temperature for 1 h in 5% nonfat dry milk in Tris-buffered saline/0.1% Tween 20 (TBST). The membranes were incubated with rabbit monoclonal anti-phospho-Tau (Ser416) (1:500; D7U25, Cell Signaling Technologies, USA) in TBST, overnight at +4C°. The membranes were than incubated with the HRP labeled secondary anti-rabbit antibody (1:2000; sc-2370, Santa Cruz, USA) in TBST for 1 h at room temperature. Three subsequent washes with 0.1% TBST were performed between each step. The signal was detected by enhanced chemiluminescence (ECL, Amersham Bioscience) and exposure of an X-ray film. All films were analyzed by densitometry using the computerized image analysis program ImageQuant ver. 5.2 (Amersham). The Ponceau S staining of the membranes served as endogenous control. The relative values of the signals (normalized to the corresponding Ponceau S staining) were determined and further statistically analyzed.

### Histological analysis

#### Preparation of tissues for histological analysis

Following the perfusion with ice-cold phosphate-buffered saline (PBS), brains were harvested, and one hemi-brain drop fixed in 4% paraformaldehyde/PBS and cryopreserved in graded sucrose solutions (10–30% w/v sucrose/PBS) for sectioning. The brains were frozen in isopentane, cooled on dry ice and stored at -80°C. Every third coronal section (20 μm thick) was collected and stored at -20°C.

#### Amyloid plaque staining

For amyloid plaque staining, Thioflavin S (ThioS) and AmyloGlo (BioSense, TR-300-AG) staining was used. ThioS was used for double staining with amyloid beta 42 antibody. Following protocol for amyloid beta 42 immunostaining, sections were washed three times in 0.01 PBS, and incubated in 0.01% ThioS solution in 50% ethanol for 8 min at RT. Stained sections were washed briefly in 80% and 96% ethanol, rinsed in distilled water and mounted onto glass slides using fluorescent mounting medium (Dako). AmyloGlo staining was used for triple labeling with ionized calcium binding adaptor molecule 1 (Iba-1) and amyloid 4g8 antibodies. Before incubation with antibodies sections were transferred into a 70% solution of ethanol for 5 minutes at RT. The slides were then rinsed in distilled water for 2 minutes. Sections were transferred for 10 min to a 1:100 of AmyloGlo dissolved in 0.9% saline solution, rinsed in 0.9% saline solution for 5 minutes, and briefly rinsed in distilled water. Following AmyloGlo staining, sections were incubated with Iba-1 and amyloid 4g8 antibodies.

### Immunohistochemical analysis

#### Immunofluorescence

Sections were blocked in 1% bovine serum albumin (BSA) in PBS for 1h in RT, incubated overnight at 4°C with primary antibodies listed in Table 1, followed by appropriate secondary antibodies (anti-mouse or anti-rabbit conjugated to Alexa 488 or 568; Invitrogen) used at the 1:250 concentration in PBS for 2h at room temperature (RT). Sections incubated in parallel without primary antibody were included as negative controls for autofluorescence and background binding of the secondary antibody. Sections were mounted with Fluorescent Mounting Medium (Dako).

**Table 1:** Primary antibodies used in this study.

antibody against	catalogue No.	host/clonality	manufacturer	Dilution
Amyloid β 42	700254	rabbit monoclonal	Invitrogen	1:500
Purified Abeta 4G8	800701	mouse monoclonal	BioLegend	1:2000
SMI31	LN#E10214GF	mouse monoclonal	Covance	1:5000
Iba-1	019–19741	rabbit polyclonal	WAKO	1:250

#### Quantitative analysis of Aβ42 clusters and SMI31-labeled DNs

Images were captured on an Axio Observer Microscope Z1 using an AxioVision 4.6 software system (Carl Zeiss, Germany) at a magnification of 5×, 10× and 40×. For quantification of Aβ42 clusters and SMI31-labeled dystrophic neurites we used available software provided by the National Institute of Health (NIH, USA)—Image J, Version 1.74. The software analyzed each cross-section. We have designated a threshold of the fluorescence intensity. When setting the limit value for analysis we have opted for the automatic value (Maximum Entropy), instead of manual adjustment, in order to perform further analysis equally in all cross-sections and to reduce faults. Based on given parameters we produced values of the surface of Aβ42 clusters. The values were further processed statistically and expressed as percentage of parietal cortex covered by Aβ42 and average surface of Aβ42 clusters.

Analyses of SMI31-positive spheroids number and % of area covered with SMI31 immunostaining were done using Image J with threshold processing (Max Entropy) and low surface area limit set to 50μm^2^. The low surface area limit was set in order to exclude SMI31-positive particles of lower surface which correspond to SMI31 immunostaining of normal (non-dystrophic) neurites. Based on given parameters we produced values of the surface of SMI31-positive spheroids number and % of area. The values were further processed statistically.

#### Confocal microscopy and image analysis of colocalization between Iba-1 and Aβ 4G8

Amyloid β and Iba-1 labelling were visualized with a Leica TSC SP8 confocal microscope (Leica Microsystems) equipped with 405/488/552 nm lasers using 40x/1.30 oil immersion lens. Images were taken with a step size of 1μm. Emission of fluorescently labelled Aβ and Iba-1 were collected sequentially. All images were taken with a 1024×1024 pixel resolution and 8-bit colour depth. Colocalization analysis was performed in ImageJ software (NIH, USA) using JACoP plugin [36] and the degree of colocalization between Iba-1 and Aβ4G8 was measured using the Pearson correlation coefficient (PCC). PCC measures the strength of a linear relationship between fluorescent intensities from the two images and produces values ranging from 1 (perfect positive correlation) to -1 (perfect inverse correlation), with 0 representing a random distribution [37].

#### Confocal microscopy and quantification of Iba1-positive microglia/macrophages

For Iba-1 imaging conditions were the same as described above. The number of Iba-1-positive microglia/macrophages were counted in layers III to VI in parietal part of the somatosensory cortex in 1 field per section under the area of the screen (400μm x 550μm) in 3 sections per animal (n = 8 animals per each group). The sections were analyzed by a researcher unaware of the treatment.

#### Confocal microscopy and quantification of plaque perimeter covered by microglia/macrophages

For Iba-1 and Amylo Glo imaging conditions were the same as described above. Confocal images of plaques selected for their similar size, location and cortical layer (layers III to VI in parietal part of the somatosensory cortex) were obtained for quantitative imaging using a a Leica TSC SP8 confocal microscope. Images were numbered randomly and blinded for analysis. All images were analyzed in ImageJ software. Five optical slices through the center of the plaque were used for analysis. On each slice, the intersection between the tips of microglia/macrophages process and the plaque perimeter were identified manually. The proportion of the plaque perimeter covered by microglia/macrophages processes was calculated by summing the arcs of plaque perimeter that colocalized with microglial/macrophage processes. For each measurement, the average of results for 5 optical slices was reported as a measurement for the individual plaque.

#### Statistical analysis

Significant differences between the data sets were determined using STATISTICA software, Ver. 6.0, StatSoft. Further statistical analysis was made using relative values. Considering that data did not meet the criteria of a normal distribution, nonparametric Man-Whitney U test was used for comparisons among two experimental groups. Statistical significance was set at P < 0.05.

## Results

### Three weeks of fish oil supplementation ameliorates neuritic dystrophy and tau hyperphosphorylation in parietal cortex of 5xFAD mice

**Fig 1:**
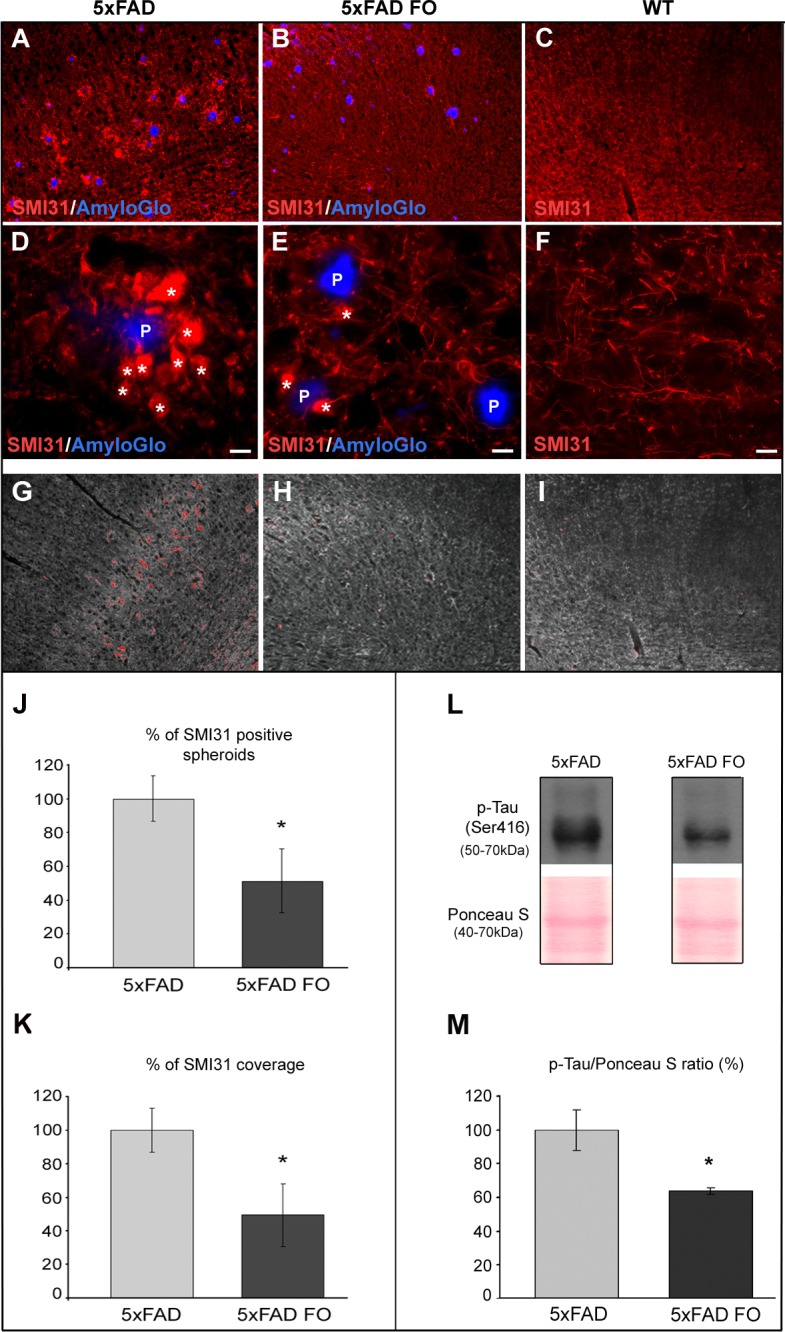
FO supplementation ameliorates neuritic dystrophy through suppression of abnormal tau hyperphosphorilation in the parietal cortex of 5xFAD mice. (A and D) Axonal dystrophies (arrows) surrounding amyloid plaques (P) in 4-month-old 5xFAD mice; (B and E) Brains of FO-supplemented 5xFAD mice showing significant suppression of dystrophic axons around plaques; (C and F) wild type mice showing absence of dystrophic neurites (G-I) Print screen of ImageJ SMI31 analysis (Max Entropy threshold). (J) Percentage of SMI31-positive spheroids larger than 50μm^2^ in untreated and FO-treated 5xFAD mice. (K) Percentage of SMI31 coverage of parietal cortex in untreated and FO-treated 5xFAD mice (low surface area limit set to 50μm^2^). (L) Representative immunoblot of p-Tau (Ser416) in untreated and FO-treated 5xFAD mice. (M) Changes in p-Tau (Ser416) protein in the cortex of 5xFAD and 5xFAD FO mice were revealed by Western blot analysis. The data represent mean ± S.E.M. value; *p < 0.05. Scale bar (D-F) - 5μm.

Swollen, dystrophic neurites surrounding amyloid plaques represent one of the hallmarks of AD pathology. Since neuritic dystrophy inevitably leads to neuronal cell death, it is of vital interest to prevent this aspect of AD pathology. In order to determine whether fish oil supplementation can affect the neuritic dystrophy we stained sections from the parietal cortex of the control and FO-treated 5xFAD mice with SMI31 antibody. In non-supplemented 5xFAD mice in the vicinity of amyloid plaques, SMI31 staining revealed swollen, dystrophic neurites (Fig 1A and 1D asterisks). However, the analysis of brain sections of FO-supplemented 5xFAD animals showed that most of the neurites have normal (non-dystrophic) appearance (Fig 1B and 1E), and are similar to neurites observed in the cortex of age-matched wild-type controls (Fig 1C and 1F).

In order to size the effect of the FO treatment, we measured the SMI31-positive spheroids number and % of SMI31 immunopositive area in the parietal cortex of untreated and FO-treated 5xFAD animals (Fig 1J). The analysis revealed that FO treatment significantly lowered the number of SMI31-positive spheroids (by 48.6%, MWU test, p = 0.03) and the percentage of the area covered by SMI31 (by 50.62%, MWU test, p = 0.018) when compared to untreated 5xFAD animals (Fig 1K). Thus, the short-term (3 weeks) fish oil supplementation is able to suppress neuritic dystrophy in the parietal cortex of 5xFAD mice.

Dystrofic neurites are characterized by abnormally hyperphosphorylated tau protein that becomes incompetent in maintaining the stability of microtubules. Western blot analysis of p-Tau (Ser416) was performed in order to determine whether fish oil supplementation suppresses neuritic dystrophy by affecting the level of tau hyperphosphorylation. The analysis revealed that the level of p-Tau (Ser416) was significantly decreased (by 36%) in FO-treated as compared to untreated 5xFAD animals (MWU, p = 0.033) (Fig 1L and 1M).

### Fish oil supplementation reduces the area covered by Aβ42 and the average size of Aβ42 clusters in the parietal cortex

**Fig 2:**
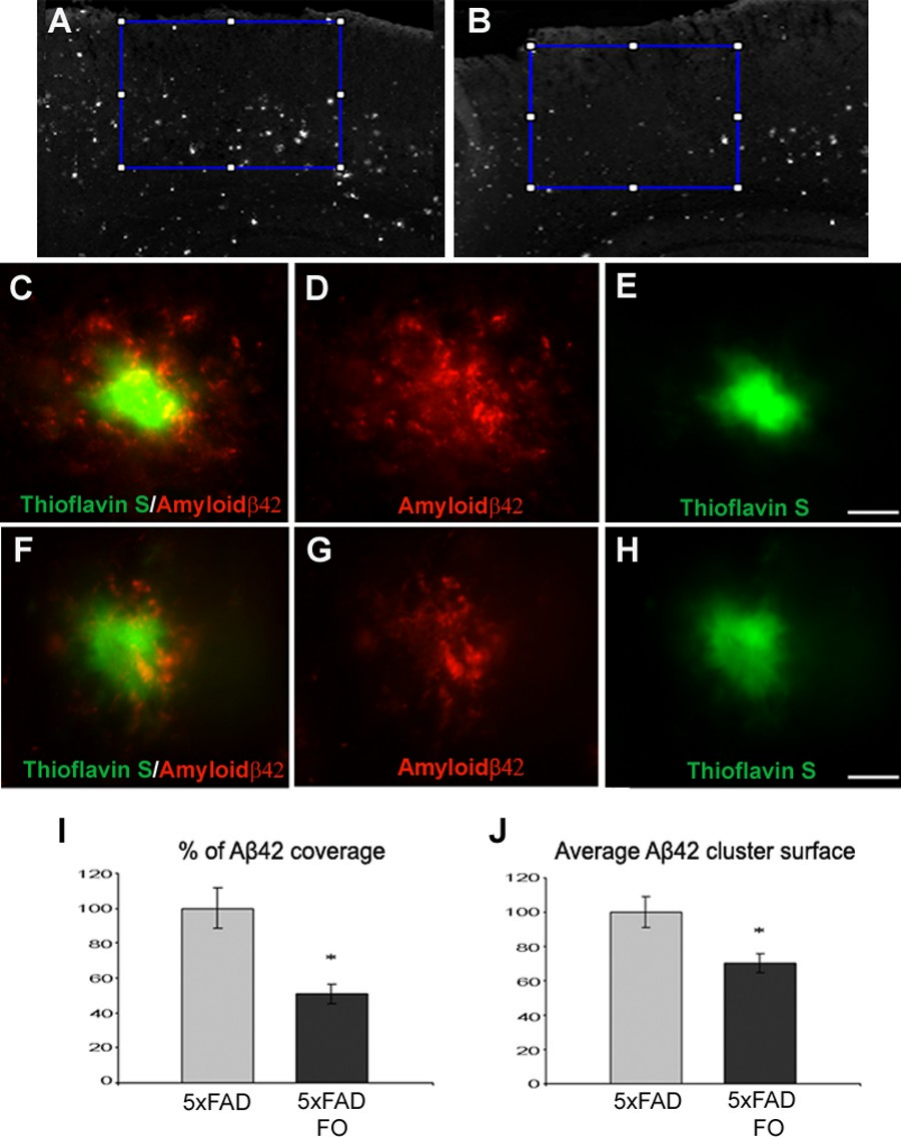
FO supplementation significantly reduces Aβ_42_ in the parietal cortex of 5xFAD mice. (A) The Aβ42 coverage of the parietal cortex in untreated and in (B) FO treated 5xFAD mice. (C-E) Representative images of Aβ42 clusters in parietal cortex of untreated and treated (F-H) 5xFAD mice. (I) Quantification of Aβ42 coverage of parietal cortex of untreated vs. treated 5xFAD mice from 8 animals for each group. Data represents mean ± s.e.m. (J) Quantification of average Aβ42 cluster surface in the parietal cortex of untreated vs. treated 5xFAD mice. Scale bar 10μm.

It is widely accepted that amyloid β (Aβ42) that surrounds amyloid plaques represents the main cause of neuritic dystrophy in Alzheimer’s pathology. Since we showed that fish oil supplementation is able to suppress neuritic dystrophy in the parietal cortex of 5xFAD mice (Fig 1), we wanted to assess whether it is able to alter the total amount of Aβ42 in this cortical region. The immunostaining of parietal cortex of control and FO-treated 5xFAD mice with anti-Aβ42 antibody (Fig 2) revealed that in both control and FO-treated 5xFAD mice, Aβ42 localization was observed mainly surrounding the plaques of insoluble amyloid β (stained with Thioflavin-S) (Fig 2C–2H). In order to size the effect of the FO treatment, we measured the surface area of the parietal cortex stained with anti-Aβ42 (Fig 2A and 2B). The analysis of surface percentage immunostained with Aβ42 revealed that FO treatment significantly lowered (by 49.14%) the area covered by Aβ42 when compared to the untreated 5xFAD animals (MWU test, p = 0.0047) (Fig 2I). Moreover, Image J analysis revealed that FO supplementation significantly reduced the average size of Aβ42 clusters (by 29.6%) in the parietal cortex of treated 5xFAD animals when compared to the untreated 5xFAD animals (MWU test, p = 0.0209) (Fig 2J).

**Fig 3:**
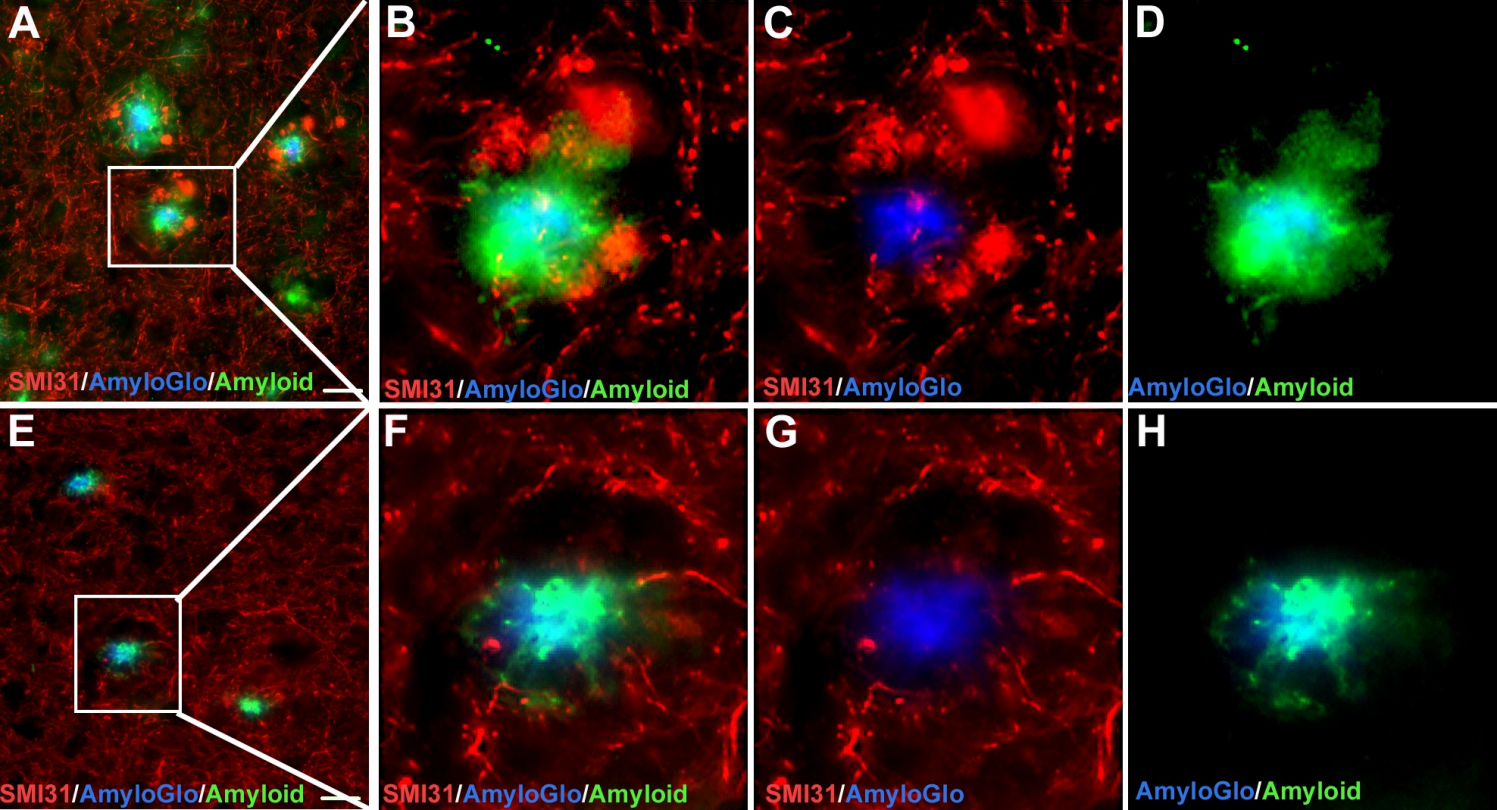
Decreased Aβ42 halo overlaps with the decreased incidence of DNs in FO treated mice. (A-D) Double staining of untreated 5xFAD mice brain sections with the 4g8 and SMI31 antibodies revealed that the areas with higher incidence of DNs display the greater total Aβ halo around the plaques (AmyloGlo+). (E-H) In the FO-treated 5xFAD animals the decreased surface of the Aβ halo overlaps with the decreased incidence of swollen, dystrophic neurites.

The Aβ42 “hot-spots” are the sites of greater neurotoxicity and we wanted to see if the decrease in Aβ42 covered surface around plaques relates to the lower occurrence of DNs. Double staining with Aβ42 and SMI31 antibodies revealed that the areas with higher incidence of DNs display the greater Aβ42 halo around the plaques (AmyloGlo+) (Fig 3A–3D). The opposite was observed in FO treated 5xFAD animals: the decreased surface of the Aβ42 halo overlaps with the decreased incidence of swollen, dystrophic neurites (Fig 3E–3H).

### Fish oil supplementation does not affect the levels of proinflammatory cytokines and phagocytosis of Aβ42 by microglia/macrophages in the parietal cortex of 5xFAD mice

**Fig 4:**
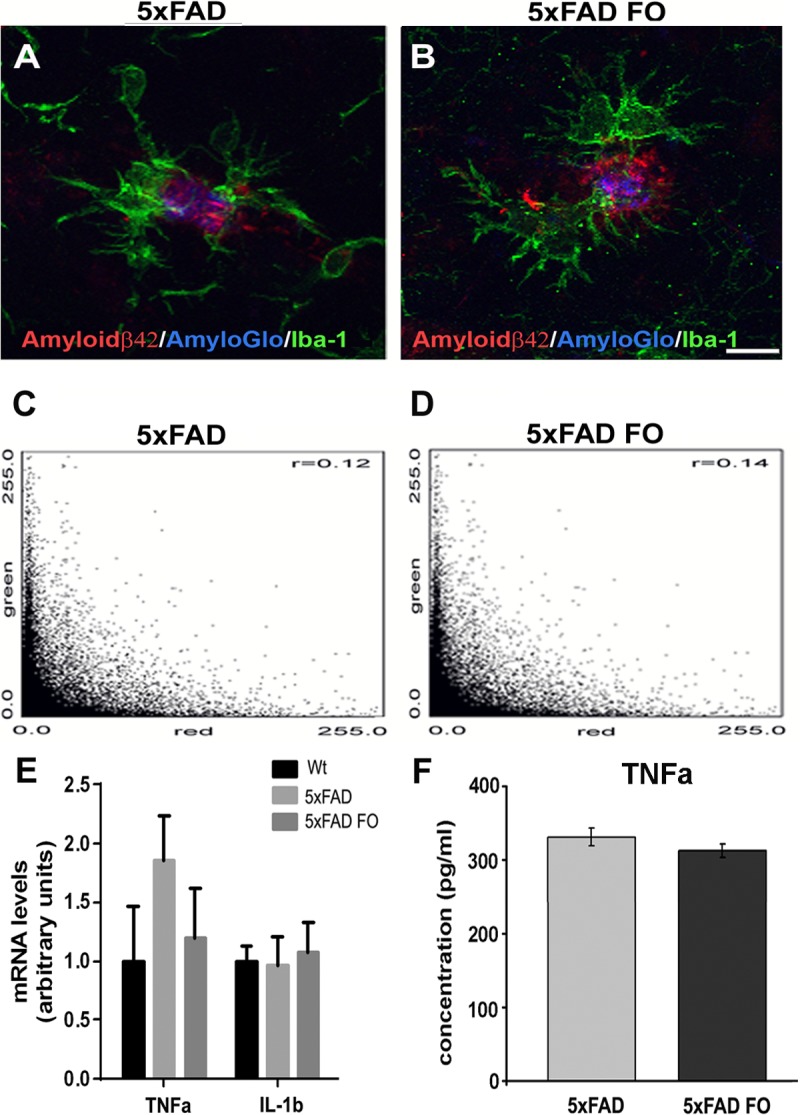
FO supplementation does not affect the phagocytic properties of microglia/macrophages and levels of proinflammatory cytokines. ((A,B) Representative confocal images showing absence of phagocytosis of Aβ42 by microglia/macrophages as assessed by measuring colocalization of Iba-1 and Aβ42 immunostaining. (C)The obtained PCC values for non-supplemented and (D) FO-supplemented animals from n>90 plaques from 8 animals for each group. (E) The RealTime PCR analysis of TNFα an IL-1β. (F) TNFa protein levels as revealed by ELISA Scale bar (A,B) - 10μm.

Since the phagocytosis of Aβ protein by microglia/macrophages has been proposed as an Aβ-lowering mechanism in AD [38, 39], we wanted to see whether the observed increase in Iba-1 + cell number correlates with the increased colocalization of Aβ42 in microglia surrounding the plaques. This information was assessed by measuring colocalization of Iba-1 and Aβ42 from the images obtained by confocal microscopy. The degree of colocalization between Iba-1 and Aβ42 was calculated using the Pearson correlation coefficient. The obtained PCC values for non-supplemented and FO-supplemented animals (r = 0.125063 and r = 0.14805, respectively) varied between 0.3 and -0.3 which is indicative of very weak to no correlation (Fig 4C and 4D) suggesting that although the FO treatment increased the number of microglia/macrophages around plaques, it did not affect their phagocytic properties.

Given the well described anti-inflammatory action of fish oil [40, 41], we next examined the expression of TNFα an IL-1β, the main proinflammatory cytokines expressed by activated microglia. The RealTime PCR analysis revealed no difference in IL-1β mRNA expression levels among Wt, 5xFAD and 5xFAD FO animals (Fig 4E). Although a tendency to alter the levels of TNFα mRNA expression was observed following FO treatment, these changes did not reach an overall statistical significance due to variation of TNFα mRNA expression levels between the samples. Moreover, no changes were observed in the TNFα protein level among 5xFAD and 5xFAD FO group (Fig 4F).

### Fish oil supplementation significantly elevates the total number of microglia/macrophages in the parietal cortex and the average number of these cells associated with amyloid plaques, accompanied by the increased plaque envelopment

**Fig 5:**
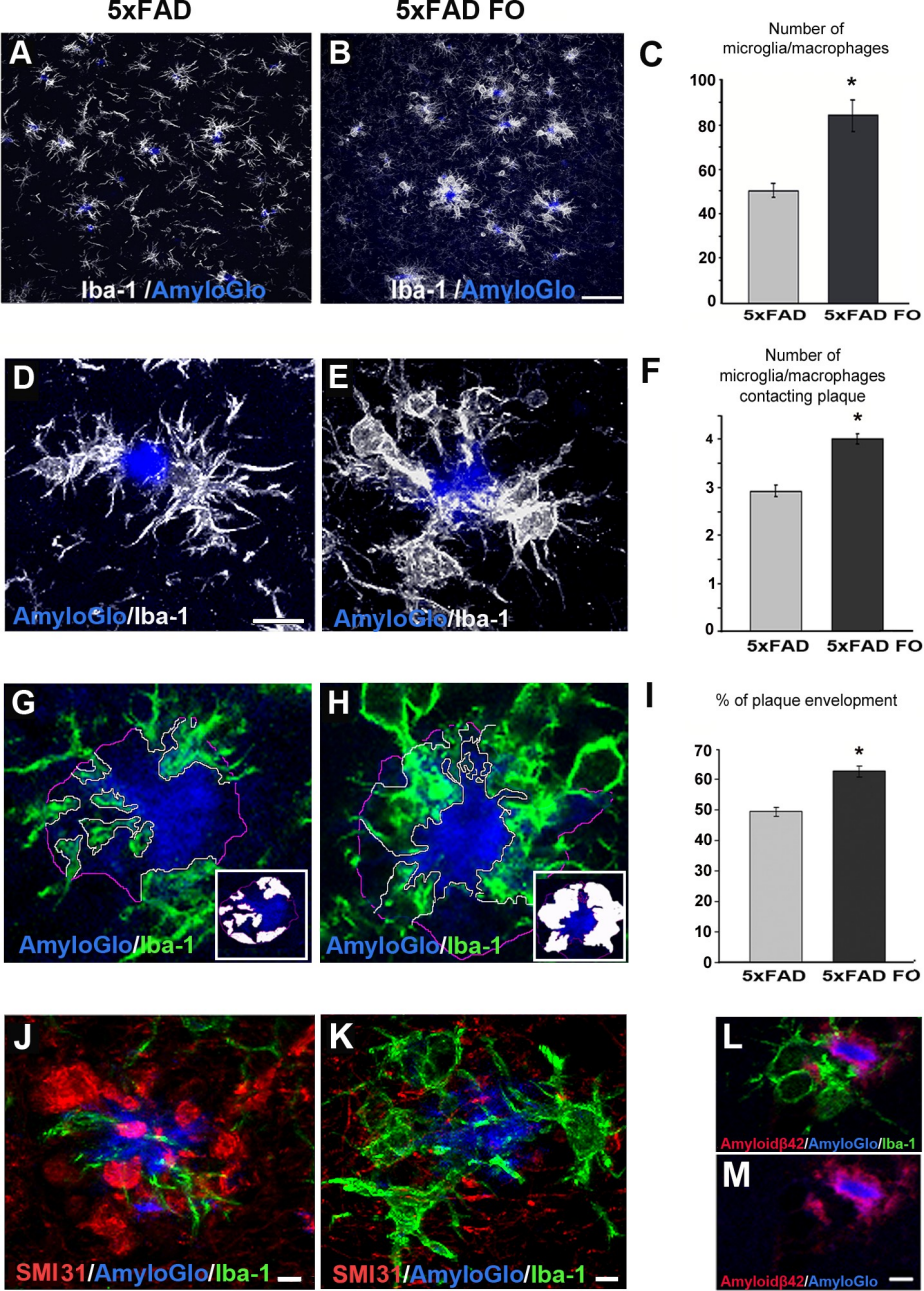
FO supplementation affects the total number of microglia/macrophages, average number of plaque associated microglia/macrophages and plaque envelopment. (A) Representative confocal images of Iba-1 immunolabeled microglia/macrophage in parietal cortex in untreated and (B) FO treated 5xFAD mouse. (C) Quantification of number of Iba-1 positive microglia/macrophages in parietal cortex of untreated vs. treated 5xFAD mice in from 8 animals for each group. Data represents mean ± s.e.m.(D) Representative confocal images of Iba-1 immunolabeled microglia/macrophages around AmyloGlo-labeled amyloid plaques in untreated and (E) FOtreated 5xFAD mice, larger magnification. (F) Quantification of number of plaque-associated microglia/macrophage in untreated vs. treated 5xFAD mice in n>90 plaques from 8 animals for each group. Data represents mean ± s.e.m. (G) Representative confocal images of Iba-1 immunolabeled microglial/macrophage processes (green) contacting AmyloGlo-labeled amyloid plaques in untreated and (H) FO-treated 5xFAD mice. (I) Microglia/macrophage coverage was quantified as the percentage of plaque perimeter contacted by microglial/macrophage processes in n>90 plaques from 8 animals for each group. Data represents mean ± s.e.m. (J,K) Representative confocal images of the degree of microglia coverage (Iba-1, green) versus extent of neuritic dystrophy (SMI31, red) around the plaque. (L,M) Representative confocal images of Iba-1 immunolabeled microglia/macrophages (green) and Aβ42 (red) mutual exclusion. Scale bar (A, B) - 50μm. Scale bar (D,E,G,H)– 10 μm. Scale bar (J,K,L-M)– 5μm.

Since ω-3 PUFAs may be protective against AD by modulating the immune response to Aβ [42] we wanted to see whether the FO treatment had an effect on the number and distribution of Iba-1 positive microglia/macrophages in 5xFAD mice. The quantification of microglia/macrophages number revealed that the FO treatment significantly increased (40.5%) the number of Iba-1+ cells in the parietal cortex of 5xFAD mice compared to the untreated mice (Fig 5A–5C) (MWU test, p = 0.00172). Namely, an average of 84 microglia/macrophages per field was detected in fish oil-supplemented animals, while the significantly lower number of 50 microglia/macrophages per field was detected in non-supplemented animals.

Considering that fish oil supplementation led to the increase in an overall number of microglia/macrophages in the parietal cortex, we next examined whether it affected the number of plaque-associated microglia/macrophages. To that end we counted a number of Iba-1+ cells with their processes pointed towards and permeated with the plaque surface of single plaques (Fig 5D and 5E). An average of 2.93 plaque-associated Iba-1+ cells per single plaque was counted in non-treated 5xFAD animals, while significantly higher number of 4.01 plaque-associated Iba-1+ cells (MWU test, p = 0.000140) was counted in the parietal cortex of fish oil-supplemented 5xFAD animals (Fig 5F). We next sought to understand whether the elevated number of microglia/macrophages aggregating around plaques also affected the degree of plaque surface coverage by processes of these immune cells (Fig 5G and 5H). Our results revealed that plaque surface coverage by microglial/macrophage processes was significantly elevated in the FO-supplemented 5xFAD animals (MWU test, p<0.00001) and amounted to 62.55% of total plaque surface, as compared to 49.32% of coverage in non-supplemented animals (Fig 5I).

Moreover, given that microglial/macrophage processes represent neuroprotective barrier [9] the increased number of plaque-associated microglia/macrophages observed in FO-supplemented mice might be the consequence of neuroprotective properties of fish oil. Namely, our data showed that plaque regions covered by microglia/macrophage had fewer dystrophic neurites than those without microglial/macrophage processes (Fig 5J and 5K). Since microglial/macrophage process distribution around the plaques is mutually exclusive with the localization of Aβ42 (Fig 5L and 5M), the increased microglial/macrophage envelopment of amyloid plaques in FO-supplemented mice might be responsible for the observed reduction of Aβ42 in the parietal cortex of this animals. Thus, these data strongly suggest that increased envelopment of amyloid plaques by microglial/macrophage processes represent a critical determinant of the neuroprotection driven by FO supplementation.

## Discussion

Neurodegeneration in AD is a process that evolves during prolonged period of time and when the first clinical symptoms occur the disease is so far advanced that it leaves little or no options of reverting damage that has been already inflicted. The onset of neuritic dystrophy appears in the early phase of pathology, the latent phase, thus presenting the window of opportunity for the potential treatments that can suppress the development of these pathological changes and disrupt the progression of the disease. The 5xFAD mouse represents a valuable model for studying the effects of different treatments on various aspects of AD related pathology as this AD mouse model develops the profound loss of cortical neurons by 12 months, with the initial structural insults appearing between 4 to 6 months of age [34, 35].

In the present study 5xFAD mice were treated for three weeks with FO in the latent phase of disease when the changes in animal behavior are not yet apparent. We demonstrated that even a short treatment with FO reduced the Aβ42 accumulation and DNs formation around the plaques, concurrently increasing the average microglial number and microglial plaque envelopment in the parietal cortex of 5xFAD mice.

So far, the progression of neuritic dystrophy has been linked to the Aβ protein accumulation around amyloid plaques in the AD brain [3, 43, 44]. It has been shown that Aβ mediates microtubule disruption via abnormal hyperphosphorylation of tau [45], and microtubule-based transport impairment that leads to the development of neuritic dystrophy and increased APP cleavage and Aβ production within these dystrophic neurites [5]. The microtubule-associated protein tau plays a major role in maintaining the normal morphology of the neurons, as it promotes microtubule assembly and stabilization. In the brain of AD patients, tau protein is abnormally hyperphosphorylated and thus become incompetent in maintaining the stability of the microtubules, directly affecting the morphology and biological functions of the neurons. The recent data from the study in 5xFAD mice [46] supports the prevailing amyloid cascade hypothesis [47], implying that hyperphosphorylation of tau is downstream of amyloid pathology, triggering the neurodegeneration and severe neuronal loss. Indeed, the anti-Aβ antibody treatment induces the reduction in the number and size of dystrophic neurites as soon as 3 days after Aβ deposits were cleared by this treatment [48]. The present study revealed that the short-term FO supplementation significantly suppressed DNs formation through the reduction of both Aβ content and tau hyperphosphorylation. This is in accordance with previous research showing that DHA increases neuronal survival by directing amyloidogenic processing of APP towards nonamyloidogenic processing, effectively reducing Aβ release [31]. In addition, DHA treatment has also been shown to improve microtubule stability and maintenance of active compensatory microtubule-associated proteins [49].

Besides the reduction of Aβ content, the anti-inflammatory action of fish oil is also well described [40, 41, 50–52], and many of its pleiotropic effects have been ascribed to this property. Numerous data indicate that DHA anti-inflammatory effects are accompanied by an increase in the phagocytic activity of microglial cells [53, 54]. Recent studies of fish-derived omega-3 supplementation in patients with mild cognitive impairment have shown polarization of macrophages to an intermediate M1-M2 phenotype that is optimal for Aβ phagocytosis and the stabilization of cognitive decline [54, 55]. However, our study showed for the first time that short-term FO supplementation neither suppresses inflammation nor enhances phagocytic properties of immune cells in the response to Aβ pathology, but it changes the behaviour of these cells prompting them to establish a physical barrier around amyloid plaques.

The classical M1 activation of macrophages is characterized by elevated pro-inflammatory cytokines and impaired phagocytic capacity [56, 57], while the M2 state is characterized by secretion of anti-inflammatory cytokines and elevated phagocytic capacity [58–60]. However, the M1/M2 activation states represent the polar extremes of myeloid cell activation. There is a large diversity and graduation of phenotypic states in peripheral monocyte-derived macrophages, in particular under conditions of chronic inflammation [61, 62]. It is increasingly recognized that responses of microglia to CNS injuries are more complex than M1 and M2 macrophage activation, and are likely modulated by the type of injury, timing and environment; possibly involving a continuum of states [63, 64].

It has been shown that activated microglia migrate to amyloid deposits early in the pathogenesis of a mouse model of AD [65]. However, *in vivo* studies monitoring plaque-microglia interaction did not observe any plaques being cleared by this microglial response [65]. This suggests that, unless further activated [66], microglia do not successfully clear plaques but instead may well restrict their growth, leading to the observed steady state of plaque size after initial formation [9, 65].

It is, therefore, possible that short-term fish oil treatment brings microglia/macrophages in a state of increased migration towards Aβ deposits enhancing the envelopment of amyloid plaques by their processes, without affecting inflammatory or phagocytic properties of these cells.

The importance of these findings is strengthened with the recent discovery that plaque micro-regions not covered by microglia represent hot-spots rich in the protofibrillar Aβ42 [5], a form of Aβ that is significantly more neurotoxic than larger Aβ aggregates[67, 68]. The accumulated microglial cells act as a barrier that reduces the uncovered plaque surface and consequently the number of hotspots of active Aβ polymerization. The microglial barrier can restrict further polymerization of the growing amyloid fibrils preventing their elongation and promoting their bending, thus shielding the surrounding neurites [5]. The strong enhancement of microglial/macrophage plaque envelopment observed in FO treated 5xFAD mice can thus represent the mechanism underlying the observed reduction of Aβ42 hotspots around plaques in the parietal cortex. The substantial neuritic dystrophy around fibrillar plaques with greater protofibrilar Aβ42 halo further highlights the necessity for the reduction of, not the number of plaques, but the amount of protofibrilar Aβ42 as the major cytotoxic component. As a rule, the overall degree of neuritic dystrophy around plaques is inversely correlated to the extent of microglia/macrophage plaque envelopment. However, the diffident reduction of DNs after anti-Aβ immunotherapy [48], regardless of a robust increase in the microglia envelopment, implies that negative effects of the immunotherapy might counteract the beneficial effects of the microglia barrier. The failure of such [69] and numerous other promising therapeutic approaches can be partly explained with their implementation in the later phase of the AD progression.

Condello and colleagues [9] emphasized that microglia envelopment was most effective around smaller plaques suggesting that microglia barrier is most prominent at the initial stages of fibrillar amyloid deposition and becomes less effective as the disease progresses. Similarly, studies of microglia ablation in later stages of amyloidosis did not show any effect on plaque number or neuritic dystrophy [70] indicating that it is increasingly difficult to treat symptoms after the disease occurrence. Therefore, the enhancement of the microglial/macrophage envelopment of the plaques and the consequential reduction of Aβ42 hotspots induced by the FO supplementation suggests a novel mechanism by which fish oil can inhibit the development of neuritic dystrophy.

This study provides evidence that neuroprotective effect of FO in the early phase of AD progression occurs through the modulation of microglia/macrophage number and behavior and underlines the necessity to elucidate the responsible molecular pathways. Several mechanisms are known to underlie omega-3-mediated neuroprotection, such as suppression of cell death, inhibition of inflammation, and promotion of neurogenesis [31]. In addition, omega-3 PUFA modulates microglia cell number and morphology in response to intracerebroventricular amyloid-β 1–40 in mice [42]. Also, documented DHA effects on lipid rafts, cholesterol homeostasis, amyloid burden and microtubule stability [12], can be responsible for the therapeutic effects of the FO supplementation. Aβ pathology is initiated at least two decades before cortical tau pathology and the onset of clinical AD symptoms[71,72], giving ample time for the preventive treatments to either delay or to ameliorate this debilitating disease. This is why the development of medications for early prevention and treatment, in accordance with the global epidemiological status, are considered a vital research goal.

## Conclusions

The present study shows for the first time that short-term FO supplementation in presymptomatic stage of AD, changes the behaviour of microglia/macrophages prompting them to establish a physical barrier around amyloid plaques. This barrier significantly suppresses DNs formation through the reduction of both Aβ content and tau hyperphosphorylation. It is interesting however, that short-term FO treatment neither suppresses inflammation nor enhances phagocytic properties of microglia/macrophages in the response to Aβ pathology, the effects most commonly attributed to the fish oil supplementation.

Revealing whether fish oil affects both microglia and macrophages in the same way, and understanding the precise mechanisms involved in this newly identified effect of fish oil, represent the future directions in elucidating its neuroprotective capacity in AD and other neurodegenerative disorders. Fish oil as a potential treatment in AD patients has a narrow window of applicability limited to the early stages of the disease. However, FO supplementation as a prophylactic intervention targeted to the enhancement of microglia/macrophage barrier may represent a promising strategy that offers effective neuroprotection.

## Abbreviations

AD: Alzheimer’s disease
Aβ: β-Amyloid
Aβ42: β-Amyloid 1–42
DNs: dystrophic neurites
FO: fish oil
FAs: fatty acids
ω-3: omega 3
PUFAs: poly unsaturated fatty acids
DHA: docosahexaenoic acid
EPA: eicosapentaenoic acid
PBS: phosphate-buffered saline
ThioS: Thioflavin S
BSA: bovine serum albumin
RT: room temperature
ON: over night
MWU-test: Man-Whitney U test
PCC: Pearson correlation coefficient
Iba-1: ionized calcium binding adaptor molecule 1
